# ANALYSIS OF BIOMECHANICAL PARAMETERS IN COLONIC ANASTOMOSIS

**DOI:** 10.1590/0102-6720201600020006

**Published:** 2016

**Authors:** Tiago Cavalcanti IWANAGA, José Lamartine de Andrade AGUIAR, Euclides Dias MARTINS-FILHO, Flávio KREIMER, Fernando Luiz SILVA-FILHO, Amanda Vasconcelos de ALBUQUERQUE

**Affiliations:** Experimental Surgery Unit, Hospital das Clinicas, Federal University of Pernambuco, Recife, PE, Brazil

**Keywords:** Surgical Wound Dehiscence, Prevention & Control, Saccharum, chemistry, Biocompatible materials, therapeutic use, Rats, Anastomosis, surgical

## Abstract

**Background:**

The use of measures in colonic anastomoses to prevent dehiscences is of great medical interest. Sugarcane molasses, which has adequate tolerability and compatibility in vivo, has not yet been tested for this purpose.

**Aim:**

To analyze the biomechanical parameters of colonic suture in rats undergoing colectomy, using sugarcane molasses polysaccharide as tape or gel.

**Methods:**

45 Wistar rats (*Rattus norvegicus albinus*) were randomized into three groups of 15 animals: irrigation of enteric sutures with 0.9% saline solution; application of sugarcane molasses polysaccharide as tape; and sugarcane molasses polysaccharide as gel. The rats underwent colon ressection, with subsequent reanastomosis using polypropylene suture; they were treated according to their respective groups. Five rats from each group were evaluated at different times after the procedure: 30, 90 and 180 days postoperatively. The following variables were evaluated: maximum rupture force, modulus of elasticity and specific deformation of maximum force.

**Results:**

The biomechanical variables among the scheduled times and treatment groups were statistically calculated. The characteristics of maximum rupture force and modulus of elasticity of the specimens remained identical, regardless of treatment with saline, polysaccharide gel or tape, and treatment time. However, it was found that the specific deformation of maximum force of the intestinal wall was higher after 180 days in the group treated with sugarcane polysaccharide gel (p=0.09).

**Conclusion:**

Compared to control, it was detected greater elasticity of the intestinal wall in mice treated with sugarcane polysaccharide gel, without changing other biomechanical characteristics, regardless of type or time of treatment.

## RESUMO

**Racional:**

A aplicação de produtos em anastomoses colônicas que possam prevenir o surgimento de deiscências são de grande interesse médico. O emprego do polissacarídeo de melaço de cana-de-açúcar (*Saccharum officinarum*), que possui adequada tolerabilidade e compatibilidade in vivo, ainda não foi testado para este fim.

**Objetivo:**

Analisar os parâmetros biomecânicos em suturas colônicas de ratos submetidos à colectomia esquerda após aplicação de fita ou gel do polissacarídeo do melaço da cana-de-açúcar no sítio cirúrgico.

**Métodos:**

Quarenta e cinco ratos (*Rattus norvegicus albinu*s da linhagem Wistar) foram sorteados em três grupos de 15 submetidos a: irrigação das suturas entéricas com soro fisiológico a 0,9%; aplicação de fita de polissacarídeo do melaço da cana-de-açúcar; e aplicação do gel do mesmo melaço. Os ratos foram submetidos à colectomia esquerda com anastomose primária, e tratados segundo os respectivos grupos. Cinco ratos de cada grupo foram avaliados em diferentes tempos após o procedimento: 30º, 90º e 180º dias de pós-operatório. Foram avaliadas as variáveis de força máxima de ruptura, módulo de elasticidade e deformação específica da força máxima.

**Resultados:**

As variáveis biomecânicas entre os tempos de coleta da pesquisa e os grupos de tratamento foram analisados estatisticamente. As características biomecânicas de força máxima de ruptura e o módulo de elasticidade do corpo de prova permaneceram idênticas, independente do tratamento com soro, fita ou gel de polissacarídeo, e do tempo de tratamento. No entanto, foi evidenciada maior deformação específica da força máxima da parede intestinal, aos 180 dias nos ratos tratados com gel de polissacarídeo de cana-de-açúcar. (p=0,09).

**Conclusão:**

Em relação ao controle, foi detectada maior elasticidade da parede intestinal nos ratos tratados com gel de polissacarídeo de cana-de-açúcar, sem alteração de outras características biomecânicas, independente do tipo ou tempo de tratamento.

## INTRODUCTION

The dehiscence of enteric anastomoses is a postoperative complication with an incidence of up to 5% in elective surgeries and 15% in emergency. Their occurrence may result in electrolyte imbalance, malnutrition, infection, sepsis and death^6^. Thus, it is critically important to study the prevention of dehiscence of the anastomosis after surgical resection, and local properties that lead to its pathogenesis.

The investigation of the healing of intestinal anastomoses in order to improve their results needs ways to quantification^2,^. In 1853, Paget introduced the measure of the tensile strength of tissues after severing and surgically repairing the tendons of rabbits; by use of rudimentary technology, he observed that the repaired segment gained strength during the postoperative period. In 1929, Howes et al systematized the measurement technique of tensile strength through a mechanical device (tensiometer) having reproducible results^14^. Recently there's been development of a computerized, high precision, mechanical method for determining biomechanical analyzes. When applied to the intestinal wall, the correlation between this method and that of burst pressure, already established in the literature, showed that the rupture measurements would be the most adequate in the research of the integrity and biological evaluation of anastomoses^15^.

In order to decrease the incidence of dehiscence of enteric anastomoses, new options and materials to be used in the manufacture of anastomoses, were researched. Studies cite the use of adhesive bio-glue, staplers and bio-fragmentable rings as causing milder local adverse reactions^7^, making for possible anastomotic protective factors. There are experimental studies on the use of tissue adhesives in colonic anastomoses, but they are still controversial, and prospective randomized clinical studies are still not available^18^.

Following this trend, the Nucleo de Cirurgia Experimental/Center of Experimental Surgery of the Federal University of Pernambuco held a series of chemical tests in order to adapt the sugarcane molasses polysaccharide (SCP), in a state of purity, to various surgical applications. It was theorized that due to promising biocompatibility findings^4,11^ and inherent properties of the material^11^, the use of SCP could reduce the incidence of dehiscence of enteric anastomoses after bowel resections or suturing.

It should be noted, however, that there are no reports in the literature of the use of SCP as a dehiscence of enteric anastomoses preventive. This study was conducted in order to analyze and compare the biomechanical parameters of sutured colonic anastomosis treated with normal saline solution, sugarcane polysaccharide tape and sugarcane polysaccharide gel. 

## METHODS

This research was approved by the Ethics Committee on Animal Use of the Federal University of Pernambuco, n ͦ 23076.056559/2012-58.

The study population consisted of adult Wistar rats (*Rattus norvegicus albinus*), with average age of 207 days (minimum 150 and maximum 245).

A prospective, randomized, bioassay trial was conducted. A total of 45 rats were randomized into three groups of 15. The animals of the three groups underwent a 2 cm long left colectomy followed by stump anastomosis using 4.0 polypropylene in simple interrupted sutures and treated according to the groups. In Group A (control) the suture was irrigated with 5 ml of normal saline solution; in Group B (tape) the suture was treated with a SGP tape encircling the anastomosis of the colon; in Group C (gel) the suture was treated with 5 ml of 1% SGP gel. Five rats from each group were evaluated at different times after the procedure, on the 30^th^, 90^th^ and 120^th^ days after surgery.

The following variables were assessed: 1) Maximum Rupture Force (MaxF), which is the maximum force applied just before the rupture of the test piece, expressed in Newtons (N); 2) modulus of elasticity (Mod Elast), which is the ratio between the applied stress and resulting deformation within the elastic limit, in which the deformation is completely reversible and proportional to the test piece tension, measured in megapascals (MPa); 3) specific deformation of the maximum force (Sp Def MaxF) is the ratio between the change of length in the test piece by application of the maximum rupture force and its initial length, measured in percent (%).

The animals were weighed and properly identified prior to the surgical procedure. They received an initial dose of 0,44 mg/kg of body weight of intra-muscular atropine sulfate. Approximately 10 minutes after application of atropine, the animals received ketamine (75 mg/kg) and xylazine (20 mg/kg), both also injected intramuscularly. The anesthesia depth for the procedures was monitored through regular respiratory rate and absence of reflexes to stimuli. During surgical procedures, 0.5 ml/min of oxygen were supplied via nasal mask.

The surgical access was obtained via an abdominal midline incision approximately 4 cm in length for dividing the skin, subcutaneous tissue, aponeurosis and peritoneum. After identification of the left colon, target site of the procedure, a 2 cm in length colectomy was performed, followed by confection of anastomosis using simple interrupted suturing technique and 4-0 polypropylene thread.

**FIGURE 1:**
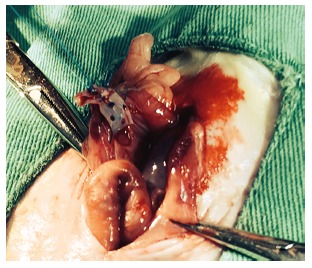
Group B animal showing polysaccharide tape of 0.5 cm wide and 3 cm long surrounding anastomotic site

In the group A animals (control group), the anastomotic suture line was irrigated with 5 ml of normal saline solution. In group B (tape), the suture line was encircled with a 0.5 cm wide and 3 cm long polysaccharide film strip, measured with the aid of a caliper, fixed in place also using 4-0 polypropylene thread (Figure 1). In animals of group C (gel) the suture line was coated with 5 ml of 1% concentration polysaccharide gel in a layer surrounding the entire anastomosis.

The animals were re-operated according with the postoperative stage, that is, in 30, 90 and 180 days. They were again weighed and anesthetized as described above. Midline laparotomy was again performed, with removal of a 3 cm long segment of the descending colon for biomechanical studies. After this procedure, the animals were euthanized with a lethal dose of intra cardiac sodium thiopental (75 mg/kg of body weight).

Biomechanical tests were performed in Polymer Science Laboratory in the Chemistry Department of the Federal University of Pernambuco, using the EMIC*^(r)^* DL500 universal biomechanical testing machine, with electronic data acquisition though the Mtest version 3 software.

The intestinal segment test piece was measured using a caliper, followed by preparation for the biomechanical and histological studies, initially by manual removal of stool content verification that the fragment encompassed all layers of the colon in both sides of the anastomosis, and throughout its whole length. The test piece was then placed in the machine, duly fixed in the prensive lugs and subjected to progressive tensile strength of up to 100N with a constant speed of 30 mm/min until breakage.

### Statistical analysis

A database was established, using a Microsoft Excel spreadsheet and exported to the Statistical Package For Social Sciences (SPSS), version 18, software. The biomechanical variables between the research's material harvest times and treatment groups were calculated using the ANOVA test, and the Kolmogorov-Smirnov test was performed initially to confirm the normality of the data. Because the data for the mortality rate was not normally distributed, the Tukey standard test was used for comparison of measures.

## RESULTS

### Biomechanical variables

In order to evaluate the biomechanical characteristics of the test piece, i.e., colon segment with anastomosis after treatments, maximum rupture force, the specific deformation of the maximum force and modulus of elasticity were measured.

It was observed that, although there was a slight variation between the treated groups, the biomechanical parameters of maximum rupture force and modulus of elasticity of the test pieces remained identical regardless of treatment with saline, tape or gel, and independent of the treatment time, 30, 90 and 180 days. However, it became evident that the biomechanical characteristic of specific deformation of the maximum force has been changed in the group treated with the gel over the different times of treatment (p=0.009, Table 1).

**TABLE 1:** Biomechanical characteristics of the specimens

Measured factor	Type of treatment			p-value
	Saline	Tape	Gel	
Max F				
30 dias	3,73 ± 0,88	3,88 ± 0,97	4,65 ± 1,30	0,386
90 dias	4,00 ± 0,41	4,02 ± 1,57	4,98 ± 1,05	0,241
180 dias	3,90 ± 0,96	3,94 ± 0,47	4,13 ± 0,71	0,898
p-valor	0,854	0,987	0,449	-
Sp Def MaxF				
30 dias	43,31 ± 13,02	64,49 ± 22,43	60,49 ± 25,53	0,295
90 dias	63,99 ± 15,91	60,28 ± 25,19	48,68 ± 9,27	0,299
180 dias	69,54 ± 30,64	62,73 ± 2,37	99,81 ± 27,87	0,137
p-valor	0,159	0,967	0,009	-
Mod Elast				
30 dias	0,43 ± 0,27	0,14 ± 0,08	0,27 ± 0,18	0,139
90 dias	0,34 ± 0,26	0,20 ± 0,18	0,33 ± 0,12	0,698
180 dias	0,33 ± 0,17	0,25 ± 0,27	0,28 ± 0,05	0,822
p-valor	0,771	0,712	0,729	-

MaxF=maximum rupture force; Sp Def MaxF=specific deformation of the maximum force; Mod elast=modulus of elasticity. Values=averages±standard deviation; p-value according to the ANOVA test

## DISCUSSION

The SGP gel and tape are exopolysaccharides, biocompatible, of water-based constitution, and low toxicity^11^. The sugarcane molasses polysaccharide has been used in various areas of experimental surgery^1^ ^,^ ^10^ ^,^ ^13^ ^,^ ^14^ ^,^ ^16^ ^,^ ^17^ ^,29^. This is the first study investigating the effect of the SGP gel and tape on their roles in the biomechanical alterations of the colon. Experimental studies have observed anastomotic alterations with different drugs^2^; however, the use of sugarcane molasses polysaccharide, was not yet known. 

The biomechanical tests analyze the strength of the anastomosis and its evolution from the time of injury and along the healing process^2^. The maximum rupture strength of anastomosis, the specific deformation of maximum force and the modulus of elasticity partially analyze the properties of the intestinal wall, because of the complexity of these structures and their non-linear viscoelastic nature^2^.

An increase in the elasticity of the structure means that the intestinal loops undergo greater strain before breaking, also contributing to the decrease of intracolonic pressure. This change may have an important role as anastomotic protective factor in certain circumstances where there is large intestinal distension, with risk of rupture.

Biomechanical tests analyze the strength of the anastomosis, its evolution from the time of injury and throughout the healing process. The maximum breaking strength, specific deformity of maximum strength and elastic modulus of an anastomosis partially analyze the properties of the intestinal wall, because of the complexity of these structures are considered viscoelastic and not linear^2^

The measure of the maximum rupture force of an anastomosis corresponds best to the biological evaluation of anastomotic healing^2^. It was found that although the average maximum force of rupture was greater in the gel treatment, the average of the comparison tests was not significant, proving that there was no SGP influence in an increased anastomotic rupture force. 

The modulus of elasticity is a fundamental parameter, since it is associated with the description of various other mechanical properties, eg the breakdown. Several researchers opt for this method of evaluation because it proved to be most accurate for measuring tissue resistance, and to accurately reflect the integrity of anastomoses^3^. Despite this, such a method has no use in the initial healing period, because until the fourth postoperative day there are no comparative changes in anastomotic resistance. Only after 14 days does the resistance increase, and that is attributed to wound healing, and not the strength of the anastomotic sutures^5^. Still regarding Mod. Elast, it was observed that the highest average found in groups of treatment was at 30 days for the saline group (average=0.43) at 90 days for gel group (average=0.33) and at 180 days for the tape group (average=0.25). Still, even considering the highest average modulus of elasticity in the described study phases, the mean comparison test was not significant in any treatment group. Meaning that the affixing of SGP in colonic anastomosis in rats also does not differ with respect to tissue resistance.

The specific deformation of the maximum force of the test piece is the ratio between the change in its length specimen when subjected to the maximum force before the break and its initial length. A higher specific deformation of the maximum force means that the colonic wall will be able to endure greater strain or distension before rupturing.

Analyzing the spec def of max force in each treatment group, it was observed that the averages comparison test was significant in only one treatment group (p=0.009 for the gel) indicating that this parameter brings an significant increase at 180 days in the group using the gel treatment.

## CONCLUSION

Compared to control, it was detected greater elasticity of the intestinal wall in mice treated with polysaccharide gel sugarcane, without changing other biomechanical characteristics, regardless of type or time of treatment.
